# A Computer Method for Pronation-Supination Assessment in Parkinson’s Disease Based on Latent Space Representations of Biomechanical Indicators

**DOI:** 10.3390/bioengineering10050588

**Published:** 2023-05-13

**Authors:** Luis Pastor Sánchez-Fernández, Alejandro Garza-Rodríguez, Luis Alejandro Sánchez-Pérez, Juan Manuel Martínez-Hernández

**Affiliations:** 1 Centro de Investigación en Computación, Instituto Politécnico Nacional, Juan de Dios Bátiz Ave., México City 07738, Mexico; 2 Electrical and Computer Engineering Department, University of Michigan, 4901 Evergreen Rd, Dearborn, MI 48128, USA; 3 Instituto Politécnico Nacional, Escuela Nacional de Medicina y Homeopatía, Guillermo Massieu 239, México City 07320, Mexico; jmmartinezh@ipn.mx

**Keywords:** artificial intelligence, machine learning, wearable sensors, Parkinson’s disease, biomechanical behavior, engineering application, medicine

## Abstract

One problem in the quantitative assessment of biomechanical impairments in Parkinson’s disease patients is the need for scalable and adaptable computing systems. This work presents a computational method that can be used for motor evaluations of pronation-supination hand movements, as described in item 3.6 of the Unified Parkinson’s Disease Rating Scale (MDS-UPDRS). The presented method can quickly adapt to new expert knowledge and includes new features that use a self-supervised training approach. The work uses wearable sensors for biomechanical measurements. We tested a machine-learning model on a dataset of 228 records with 20 indicators from 57 PD patients and eight healthy control subjects. The test dataset’s experimental results show that the method’s precision rates for the pronation and supination classification task achieved up to 89% accuracy, and the F1-scores were higher than 88% in most categories. The scores present a root mean squared error of 0.28 when compared to expert clinician scores. The paper provides detailed results for pronation-supination hand movement evaluations using a new analysis method when compared to the other methods mentioned in the literature. Furthermore, the proposal consists of a scalable and adaptable model that includes expert knowledge and affectations not covered in the MDS-UPDRS for a more in-depth evaluation.

## 1. Introduction

Parkinson’s disease (PD) is a neurodegenerative disorder that predominantly affects dopamine-producing neurons, with symptoms that develop slowly over the years. For example, tremors, bradykinesia, limb rigidity, gait and balance problems, and other unrelated symptoms to movements, such as depression and cognitive impairment, can be present [1]. Currently, all therapies used for PD improve the symptoms without slowing or halting disease progression.

One of the most widely accepted clinical rating scales for PD progression is the “Unified Parkinson’s Disease Rating Scale” from the Movement Disorder Society (MDS-UPDRS) [2]. The MDS-UPDRS has been used in different works [3,4,5,6] as a guideline to develop several computational tools to objectively assess the progression of PD motor symptoms. Some motor affectations that are used to determine the severity of the disease are gait, tremors, and bradykinesia, as presented in [7,8,9,10,11]. Although, these do not include an analysis of the pronation-supination hand movements.

Several artificial intelligence methods have assessed chronic diseases [12], such as PD and diabetes. In the case of PD, in the work presented by [13], an automatic non-invasive method is used to identify Parkinson’s-related gait. The previously mentioned work uses a combination of wavelet analysis and support vector machines (SVMs), reporting a classification accuracy of 90.32%.

Other works present different neural network architectures to assess motor symptoms, such as convolutional neural networks (CNNs) for classifying the severity of PD patients [4,14,15]. The first and second studies present 85% and 95% accuracy, respectively. However, they depend on the convolutional filters of CNNs for feature extraction and use methods like handwritten dynamics, which clinical experts find difficult to interpret most of the time. Meanwhile, in the third study, classification performance improved from 77.54% to 86.88% after a data augmentation process.

In [16], motor, nonmotor, and imaging features were extracted using unsupervised and supervised methods. However, this work explicitly investigates optimal feature selection for the robust identification of PD subtypes. Other studies, such as [17], developed a sex-specific and age-dependent classification method to diagnose Parkinson’s disease using the online handwriting of patients with PD with an accuracy of 83.75% for females and 79.55% for an old-age classifier.

The MDS-UPDRS [2] presents instructions for assessing pronation and supination hand movements (item 3.6). Each hand is evaluated separately. The patient must extend the arm out in front of his/her body with their palms facing down, then turn the palm up and down alternately 10 times as fast and as fully as possible. The exercise evaluation includes speed, amplitude, hesitations, halts, and decrementing amplitude. The movement assessments are ***Normal*** if there are no problems, and ***Slight***, when any of the following behaviors occur: (a) the regular rhythm is broken with one or two interruptions or hesitations; (b) slight slowing; (c) amplitude decrements are observed close to the end of the sequence. ***Mild*** is used if any of the following occur: (a) three to five interruptions during the movements; (b) mild slowing; (c) amplitude decrements are observed halfway into the sequence. Moderate is used (a) when there are more than five interruptions during the movement or at least one longer arrest (freeze), (b) moderate slowing, and (c) amplitude decrements are observed starting after the first supination-pronation sequence. ***Severe*** is used if the patient cannot or can only barely perform the task due to slowness, interruptions, or decrements.

Nevertheless, the patients can present affectations not evaluated by the UPDRS, such as a wobble during the hand movements and the speed change rate during different stages of the exercise. These affections are very difficult to assess visually, and it is even more complex to monitor their evolutions in regular patient consultations. In this sense, in [5], 12 characteristics related to the above behavior were extracted, and a feature selection was applied.

Machine learning has been used for tremor severity quantification in Parkinson’s disease and upper limb motor preclinical assessment and diagnosis using voice features [4,9,18]. However, pronation-supination hand movements have not been evaluated.

In [19,20], fuzzy inference models were used to assess pronation-supination hand movements based on the MDS-UPDRS. These works calculated biomechanical indicators from accelerometers, gyroscopes, and magnetometer signals. The indicators were quantified through other feature engineering methods to represent several motor impairments considering more than 350 measurements from 57 PD patients and 10 healthy control subjects. The modeling of the expert’s knowledge through fuzzy logic has proved to be successful as they provide a score that is easy to interpret by the expert clinicians. In [20], two different fuzzy inference models were combined with a decision-making process for a complete assessment. 

As mentioned, fuzzy inference models present the advantage of decreasing ambiguity and interpretability regarding people of different backgrounds [21], in particular, for deciding when medical specialists should be involved in the design and validation of computerized expert systems. However, when many indicators exist, creating fuzzy systems (membership functions and fuzzy rules) presents significant complexity. Scalability is an inconvenience in this model type. If the criteria to evaluate the PD patients change, the membership functions and the fuzzy rules must be redesigned. 

In [22], several parameters from upper limb motion were extracted, including pronation and supination hand movements. Even though some of the presented parameters are mentioned in the MDS-UPDRS, they only classify healthy control subjects and patients with PD. 

The current proposal addresses the scalability and adaptability issue with a self-supervised training approach, maintaining the result’s interpretability by expert clinicians. In this sense, our use case focuses on the motor impairments of patients, given that these affectations can be objectively measured. The proposed method generates latent space representations in an unsupervised manner; this addresses the scalability issue as new biomechanical features may appear over time. Another advantage is that the method can easily adapt to the recent expert’s evaluation criteria by relabeling the original observations without retraining the model using a lazy learning method.

The method consists of three modules. The first module is an auto-encoder, a type of neural network with an encoder-decoder architecture. This neural network can efficiently compress data into a low dimension (latent space representations) and find complex relationships in the data. In order to achieve this, the encoder transforms the input (high-dimensional data) to a lower dimension (latent space), and then the decoder can reconstruct the same inputs [23,24,25]. The second module consists of the labeling of these latent space representations. In order to accomplish this, experts give a score to the original observations of each patient according to the MDS-UPDRS guidelines [2]. These scores, which are considered so that the expert’s knowledge can be integrated, can be easily adjusted by re-evaluating the observations. In the third module, a final evaluation is given for new patients by considering the distance between the computed latent space representations and the new query point.

This paper includes a Materials and Methods section with a summary of the biomechanical indicators and the detailed design of the unsupervised and supervised stages. The Results section offers the obtained scores, and subsequently, the Discussion and Conclusions follow.

## 2. Materials and Methods

A summary of the biomechanical indicators used in this work is presented in this section. The dataset consists of 228 pronation-supination hand movement records from 57 PD patients and eight healthy control subjects (some patients have more than one measurement in a span from 1 to 6 months, Table 1) [19]. Each record consists of measurements acquired by inertial measurement units (IMU) while the patient performed pronation and supination hand movements. Two previously calibrated units were placed on the dorsal side of both hands [19]. Data acquisition was carried out via virtual instrumentation software, while self-supervised model implementation was performed with the help of the *Keras,* Sci-kit-Learn [26], and Python packages.

**Figure 1:**
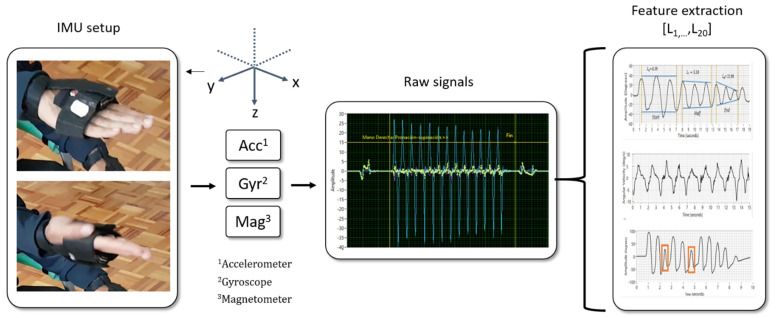
Biomechanical indicators extraction in two previously published works by our team.

As depicted in Figure 1, each measurement was acquired wirelessly by a computer connected to the IMU, recording movements on the *x*, *y*, and *z*-axis. Each IMU consists of an accelerometer, a gyroscope, and a magnetometer [19,20]. The measurements were acquired at a rate of 50 samples per second; this allowed an appropriate discretization of the continuous signals [27].

Over the years, an increasing number of biomechanical features have been proposed to describe motor impairments. According to the MDS-UPDRS and expert clinicians, several elements must be considered to rate pronation-supination hand movements, like amplitude, speed, hesitations, halts, and decrementing amplitudes. 

**Figure 2:**
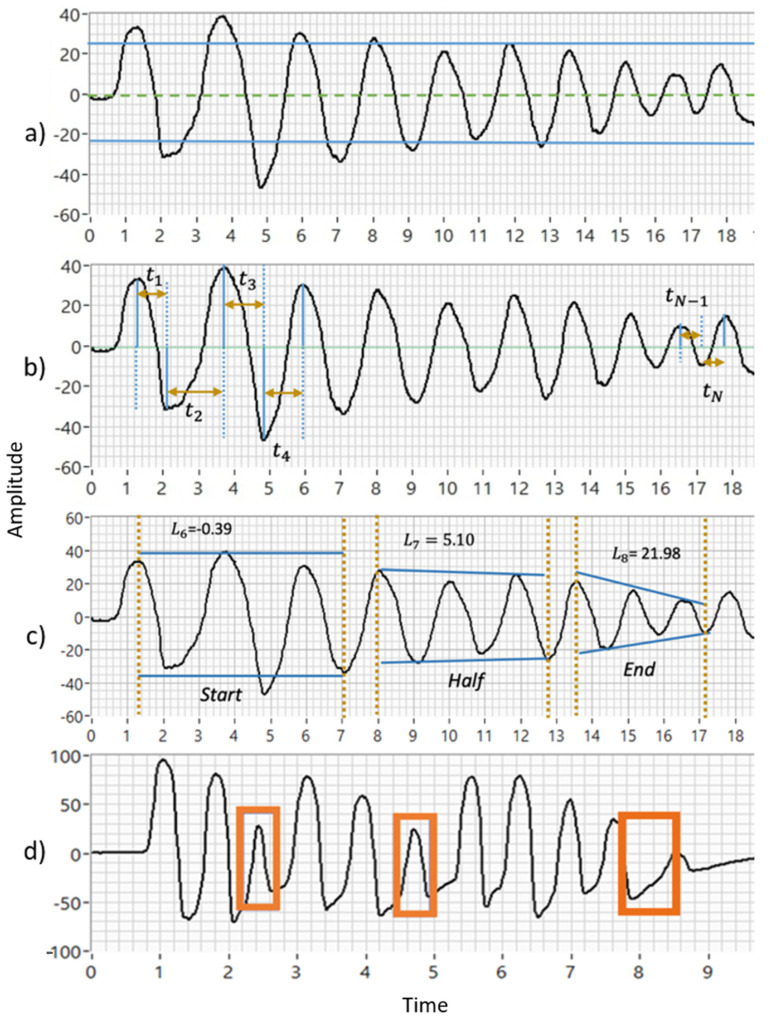
Illustration to obtain the biomechanical features that follow MDS-UPDRS guidelines ($L_{1–8}$): (**a**) mean and standard deviation of amplitude ($L_{1–2})$. (**b**) Mean and standard deviation of speed ${(L}_{3–4})$. (**c**) Decrements in amplitude during three exercise stages ${(L}_{6–8})$. (**d**) Halts and hesitations ${(L}_{5})$. Time is seconds, and other measurement units, are shown in Table 2.

Our team calculated eight indicators to evaluate motor impairments that are included in the MDS-UPDRS [19]. Their calculations are based on the hand movement behavior, illustrated graphically in Figure 2. For example, the mean and standard deviation of amplitude and speed are in Figure 2a,b. The decrements in amplitude during the three exercise stages are shown in Figure 2c (Start, Half, and End), and the halts and hesitations are illustrated in Figure 2d. Details of how these biomechanical indicators were computed are deeply explained in [19].

**Figure 3:**
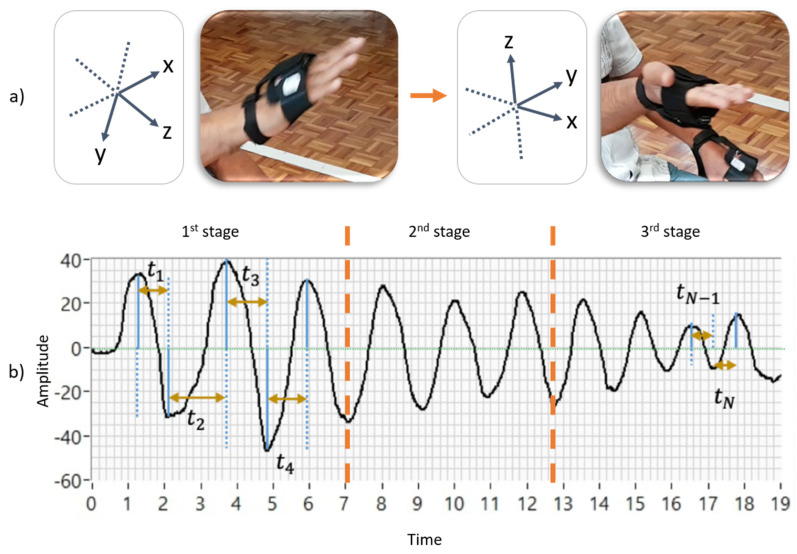
Illustration of the behavior of pronation and supination hand movements regarding biomechanical characteristics not included in the MDS-UPDRS guidelines: $L_{9}$: unsteady oscillation in the *x*, *y* & *z* axes; $L_{10}$: unsteady oscillation in the *x* and *y* axes; $L_{11}$: median amplitude; $L_{12}$: interquartile range of amplitude; $L_{13}$: median velocity; $L_{14}$: interquartile range of velocity; $L_{15–17}$: rate of velocity decrement in the sequence’s first, second and last stage, respectively; $L_{18–20}$: the slope of velocity decrement in the sequence’s first, second and last stages, respectively (calculation details in [20]). Time is seconds, and other measurement units are in Table 2. (**a**) An unsteady oscillation during hand movements (**b**) The quantify speed decrements for the three different ex-ercise stages.

In [20], an extended method evaluates the pronation and supination hand movements with additional indicators; Table 2 presents 20 indicators organized based on the characteristics of amplitude, velocity, and anomalies during the movement sequence ($L_{1–20}$). The 12 new indicators, with respect to [19], were created to evaluate the motor impairments not included in the MDS-UPDRS guidelines. For example, some visible affections, such as a wobble in the upper limbs, can be described as an unsteady oscillation during hand movements (Figure 3a), which frequently increases as the exercise time elapses. The ideas exchanged between specialists during the PD patients’ motor assessments supported this contribution. Some patients present behaviors that are not mentioned in the literature. In this sense, the variation in each axis pointing direction was quantified into two different features following an algorithm based on quaternions [28,29] and by using the triaxial dynamic acceleration and vector calculation [11]. The median of the amplitude and velocity and their interquartile ranges were computed to avoid the effect of possible outliers during these exercises. Additionally, the indicators that quantify speed decrements for the three different exercise stages (Figure 3b) were obtained. The calculation details of these new 12 biomechanical indicators are deeply explained in [20].

The new proposal creates a self-supervised adaptive model encompassing supervised and unsupervised learning methods. The task of this model is to learn good representations in the latent space derived from the original biomechanical features. These new representations resulted from transforming the 20 initial biomechanical indicators depicted in Table 2, i.e., a dimensionality reduction in the original dataset. Subsequently, a lazy learning algorithm gave a score based on the MDS-UPDRS guidelines.

The proposed method was divided into three core modules: the first consisted of a neural network to learn efficient data representations from the original biomechanical indicators. The second module included integrating the experts’ knowledge as part of the assessment of each PD patient. The last module corresponded to a supervised approach whereby patients were evaluated on a discrete and continuous scale following the MDS-UPDRS guidelines. 

**Figure 4:**
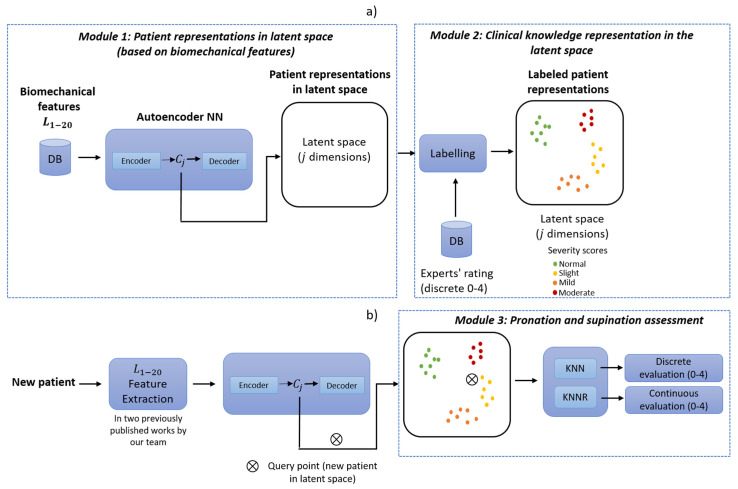
Method overview: (**a**) training phase; (**b**) deployment phase. KNN: K-nearest-neighbor and KNNR: K-nearest neighbor regression.

This method was also divided into two stages: *Training* (Figure 4a) and *deployment* (Figure 4b). *Training* refers to finding the model’s parameters, and *deployment* refers to using the method in a clinical environment. The training phase transforms each patient into the latent space based on their motor impairments. Expert clinicians then label each observation (patient performing a pronation-supination motor examination exercise) following the MDS-UPDRS guidelines. 

For expert clinicians to provide their assessment (labels), they watched previously recorded sessions of each patient performing the pronation and supination hand movements. All the evaluations were carried out following the MDS-UPDRS guidelines. The results were clusters of labeled patients in the latent space, as shown in Figure 4a. Details of the interaction between Module 1 and Module 2 are given in Section 2.1 and Section 2.2.

A tabular summary of expert clinicians’ scores is depicted in Table 3. Overall, the expert assessments had an agreement of 70–74%. Therefore, we can tell that each expert clinician tends to give a similar score for the pronation and supination movements.

Table 2 presents the 20 biomechanical indicators used to assess pronation and supination hand movements. They are organized based on characteristics of amplitude, velocity, and anomalies during the movement sequence.

An overview of the training phase in the proposed method is depicted in Figure 4a. In this method, the biomechanical features $L_{1–20}$ are used as inputs to enable the auto-encoder to learn efficient data representations in an unsupervised manner (Module 1). In Module 2, the examiners label each original observation; the expert’s knowledge is included following the MDS-UPDRS guidelines. Finally, in Module 3, a query point (new patient) in the latent space is used as input for a distance-based algorithm (K-nearest-neighbor (KNN) and K-nearest neighbors regression (KNNR)) to obtain a discrete and continuous evaluation, respectively, based on the severity of each patient in a *deployment phase*. KNN is based on the initial ideas of Evelyn Fix and Joseph Hodges [30]. 

As shown in Figure 4b, a new patient undergoes a feature extraction process for the acquired raw signals during the pronation-supination hand movements. After that, the extracted features go through the trained auto-encoder to generate their representation in the latent space. Lastly, KNN and KNNR are used to determine a discrete and a continuous score based on the closest neighbors in the latent space. 

The three modules mentioned above were implemented in Python using open-source platforms for machine learning. All the supplementary materials (data, code, and models) are available upon reasonable request to the authors.

### 2.1. Module 1: Patient Representations in Latent Space (Based on Biomechanical Features)

Auto-encoders represent a neural network that aims to compress the input into a lower dimension (latent space) and reconstruct the output from this latent space. For example, they are used for different applications [31,32]. 

Based on an auto-encoder, Module 1 automatically generates a representation of each observation in the latent space. The 20 biomechanical features ($L_{1–20}$) are used to feed the auto-encoder, as shown in Figure 4a.

**Figure 5:**
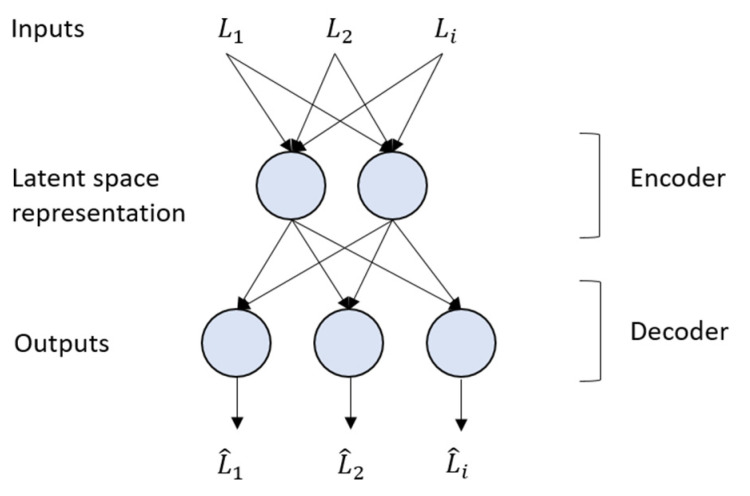
Auto-encoder architecture.

An auto-encoder topology is always composed of two parts. In our case, the first part (encoder or recognition network) converts the input to a latent representation, as depicted in Figure 5. In this work, this part consists of an input layer l composed of 20 biomechanical indicators ($l\in R^{m},m=20)$ and a hidden layer, h, of size n, where n < m. The hidden layer with fewer neurons forces the network to learn a compact representation of the original inputs. These hidden neuron outputs (representing the embedding or location of each patient in the latent space) can be interpreted as complex nonlinear combinations of the original features. Although the latent representations are learned automatically by the encoder without any supervision by humans, they are extracted from the established biomechanical features $L_{1–20}$. The use of the auto-encoder allows for finding those complex relationships automatically.

The output of this hidden layer is the latent space representation and is defined as:

(1) $$f:l\in R^{m}⟼h\in R^{n}$$

where

(2) $${h_{j}≔f}_{j}\left(l\right)=\phi_{e}\left(\theta_{j}^{T}l+b_{j}\right),j=1,2,\ldots ,n$$

where

$\phi_{e}$: is the activation function;
$\theta_{j}^{T}$: is the weights vector;
l: is the input vector.

The second part of the auto-encoder converts the internal representations to the network’s outputs. In this work, this part consisted of using the hidden layer outputs, h, as inputs to the output layer, $\hat{l}$, which then created the reconstructions, ${\hat{L}}_{i}$, of the original inputs, as seen in Figure 5. The decoder can be defined as

(3) $$g:h\in R^{n}⟼\hat{l}\in R^{m}$$

where

(4) $$g_{k}\left(h\right)=\phi_{d}\left({\theta^{\prime }}_{k}^{T}h+b_{k}\right),\;\;\;k=1,2,\ldots ,m$$

being:

$\phi_{d}:$ the activation function.
${\theta^{\prime }}_{k}^{T}:$ the weights vector.
h*:* the latent representations vector.

This functionality of the auto-encoders is also used to determine auto-encoder performance, i.e., the biomechanical features used as inputs for the encoder $L_{i}$ should be very similar to the reconstructions ${\hat{L}}_{i}$ generated by the decoder. 

As an initial approximation, the auto-encoder was organized into two parts, as mentioned before. The encoder and the decoder are regular sequential models with a single dense layer each. In total, three layers were used in the neural network architecture. The task of training is to estimate the weights $\Theta ≔[\theta_{1},\ldots ,\theta_{n}]$ and $\Theta^{\prime }≔[\theta_{1}^{\prime },\ldots ,\theta_{m}^{\prime }]$; these parameters are estimated, so the reconstruction error $e=L_{i}-{\hat{L}}_{i}$ is minimal.

Initially, all layers used the *relu* (rectified linear unit) activation function, as it is a common standard with neural networks. Additionally, the mean squared error and binary cross-entropy loss functions were tested initially. Auto-encoder optimization and its hyperparameter tuning are explained in Section 2.4.

A comparison with a popular approach for data transformation was contemplated as another approximation to determine an acceptable auto-encoder architecture. It is well known that the principal component analysis (PCA) can be compared to auto-encoders when their neurons have linear activation functions [33]. PCA, also commonly used for dimensionality reduction, offers a simple approach to determine a smaller number of components needed to reconstruct the input without losing too much information. This approach selects the number of principal components that explain some predefined percentage of the variance, usually between 90 and 95% [34]. 

In this sense, the variance of each component was computed, and the first ten principal components added up to 94% of the total variance, which gives a notion of the architecture needed for the auto-encoder. However, as mentioned before, PCA only performs linear transformations. In contrast, auto-encoders have the versatility to perform nonlinear transformations, which will help with more complex data like the biomechanical indicators of this work.

### 2.2. Module 2: Clinical Knowledge Representation in the Latent Space

**Figure 6:**
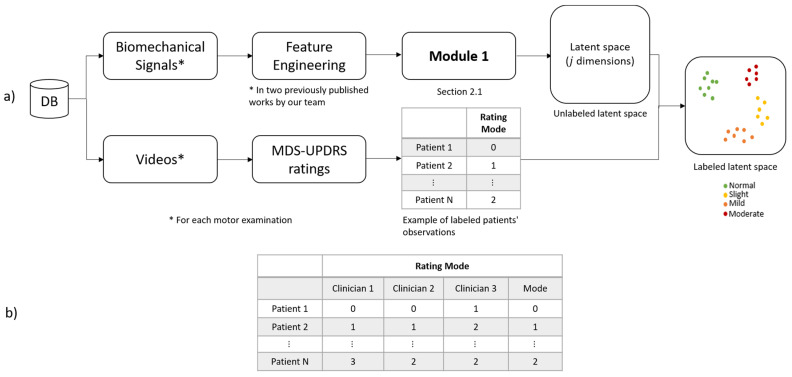
Integration of expert clinicians’ knowledge. Feature Engineering: in two previously published works by our team. (**a**) Two paths (**b**) Rating Mode.

Module 2 was used to integrate the experts’ knowledge, as depicted in Figure 4a). Two paths were followed (see Figure 6) to obtain a labeled representation of each patient in the latent space.

The first one starts with acquiring the raw signals (biomechanical signal), followed by the feature extraction process (feature engineering) presented by [19,20]. Once the features were extracted, their representations in the latent space were generated, as stated in Section 2.1. 

In the second path of Figure 6, expert clinicians evaluated recorded videos of each patient’s pronation-supination movements. Scores by the expert clinicians were given following the MDS-UPDRS guidelines (*Rating Mode*). Subsequently, the assessment (*Rating Mode*) of each patient was mapped to the latent space representation $h_{j=\{1,\ldots ,10\}}$ generated by the encoder. This resulted in labeled representations of each patient in the latent space (*Labeled latent space*). 

For the evaluation, three expert clinicians participated in the motor assessment of patients with D. Each expert gave a score according to their judgment. In order to incorporate their scores into the unlabeled latent space, the mode of the three scores was computed and mapped to each latent space representation.

It is important to mention that the expert’s knowledge integrated into this method can always be changed. This means that if a medical institution wants to use its criteria for the assessment, the experts only need to re-evaluate each PD patient, which can be easily accomplished as all motor explorations are recorded. Consequently, the method can continuously adapt to new expert criteria without redesigning the neural network architecture. 

In the same way, if medical institutions do not want to change the criteria for assessing the severity of patients for any reason. The proposed method can always be used given that the expert knowledge currently used is from three expert examiners endorsed by the MDS-UPDRS, which gives the benefit of standard criteria for evaluation.

### 2.3. Module 3: Pronation and Supination Assessment

For our use case, KNN was selected due to its advantages. The KNN algorithm is considered a lazy learning algorithm; this means that it has no explicit training step, and all the work happens during prediction with a time complexity of O(kn). This is particularly helpful because since there is no explicit training step, as we keep adding new data to the dataset, the prediction is adjusted without retraining a new model, which helps with the adaptability and scalability of our work.

The goal of module 3 was obtaining a discrete and continuous evaluation in a clinical environment, as depicted in Figure 4b. In order to achieve this, a query point (new patient) in the latent space is used as input for the distance-based learning algorithm KNN and KNNR. Using the labeled latent space representations $h_{j}$ as inputs, a discrete and continuous evaluation for a new patient can be determined. 

For the discrete evaluation, the KNN algorithm [35] was used in a classifier configuration, and the target is the mode of all the experts’ assessments computed in Section 2.2. New experts can add knowledge without modifying the auto-encoder to generate new latent space representations. To determine the class of a new query point q (new patient), KNN calculated the distance between the labeled data points and q via Equation (5).

(5) $$d\left(q,p\right)=\sqrt{\sum_{i=1}^{n}{\left(q_{i}-p_{i}\right)}^{2}}$$

where

$d\left(q,p\right):$ is the Euclidean distance between data points;
$q_{i}:$ is the query point (new patient);
$p_{i}$*: labeled* data points in latent space representation.

The results for the KNN as a classifier are depicted in Table 4. *Recall or Sensitivity* is the number of true positives divided by the total number of actual instances of the class. *Recall* is a performance metric that measures the ability of a model to correctly identify all relevant instances of a class. It is calculated by dividing the number of true positives (correctly identified instances of the class) by the total number of actual instances of the class [26]; in our case, PD patients whose severity stage was correctly identified by our model, including the false negatives (instances incorrectly classified as not belonging to the specific disease stage).

*Precision* is the number of true positives divided by the number of instances identified as belonging to the class.

The *F*1 *score* is a measure that combines *Precision* and *Recall* into a single score. The harmonic mean of *Precision* and *Recall* ranges from 0 to 1, with higher values indicating better performance. It is calculated as 2 ∗ (*Precision* ∗ *Recall*)/(*Precision* + *Recall*).

Categories 0 and 1 present a regular performance for a *normal* and a *slight* stage of the disease. *Mild* and *moderate* (2 and 3) have poor performance. The reason for these poor results is the low number of observations of these categories, leading the model to give more weight to those with higher observations. Further model tuning is later addressed in Section 2.4.

KNN can be used in cases where the data labels are continuous rather than discrete variables; the KNNR configuration [36,37] is used for these circumstances. In our case, the work presented by [20], a continuous score was computed by a decision-making process. In KNNR, the distance from q to the k-nearest data points was computed again by Equation (5). After that, the arithmetic mean of the measured distances was calculated and used as a prediction.

After obtaining the predictions of the test set, they were compared to the results presented in [20] using the determination coefficient ($R^{2}$) of Equation (6) and the mean squared error (*MSE*) of Equation (7). Table 5 depicts the results of the two metrics. Both metrics present acceptable results, with *MSE* being a small number and $R^{2}$ close to one.

(6) $$R^{2}=1-\frac{{ss}_{res}}{{ss}_{tot}}$$

where

${ss}_{res}:$ is the sum of squares of residual errors;
${ss}_{tot}:$ is the total sum of the errors.

(7) $$MSE\left(L,\hat{L}\right)=\frac{1}{n}\sum_{i=0}^{n}{\left(y_{i}-{\hat{y}}_{i}\right)}^{2}$$

where

n: is the number of elements;
$y_{i}:$ represents the predictions of KNNR;
${\hat{y}}_{i}:$ represents the values of the decision-making process.

### 2.4. Module Optimization and Hyperparameter Tuning

Several methods were used to optimize the auto-encoder and the assessment estimation, from over-sampling the original observations to hyperparameter tuning via trying different combinations, which are addressed in the following subsections.

#### 2.4.1. Dataset Over-Sampling

The studied dataset consisted of 228 records with 20 different biomechanical indicators and the scores of three expert clinicians, following the MDS-UPDRS guidelines. However, an imbalance problem in our dataset was present, as is commonly presented in real-life scenarios.

Considering the mode of the three expert ratings, the proportion of category 1 (slight stage of the disease) was the biggest one, with more than half of the entire measurements, followed by category 0, which represents the patients with minimal motor affectations or control subjects. The following categories 2 and 3 represented patients with moderate and severe motor symptoms and represented around a fifth of the total measurements, as shown in Table 4. 

As the literature states, class-imbalanced data leads to bad prediction models, and most machine learning methods tend to perform poorly in minority class examples [36,38]. In this sense, one of the most common methods used to address this issue is the *synthetic minority oversampling technique* (SMOTE) [36]. 

This oversampling method was used to create synthetic data points of all the biomechanical indicators $L_{1–20}$ for categories 0 (*normal*), 2 (*mild*), and 3 (*moderate*) in the original dataset; the original and resulting proportions are depicted in Table 6. After the oversampling method, 480 records were used in the dataset.

**Figure 7:**
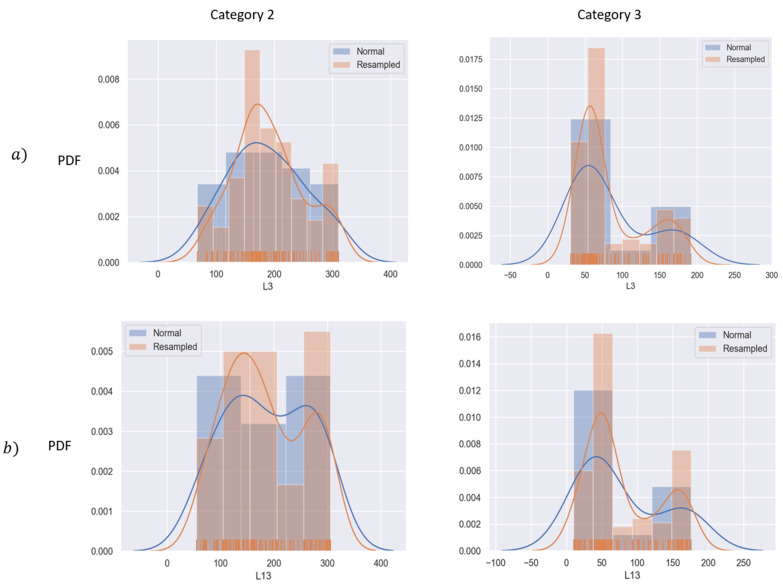
Probability density function (PDF) before and after the oversampling technique. (**a**) Mean velocity; (**b**) median velocity.

SMOTE was preferred over other oversampling techniques, such as random oversampling, due to the advantage that *the new data generated preserve the original data distribution of each feature*, as depicted in Figure 7, where the distributions of the mean velocity and median velocity are shown in blue and the resampled features in orange for patients in stages 2 and 3 of the disease.

SMOTE synthetically generates new instances between existing instances. Specifically, a random example from a minority category is first chosen. Then the *k* of the nearest neighbors is found. Finally, a randomly selected neighbor is chosen, and a synthetic example is created at a randomly selected point between the two points. This is important because overfitting is avoided, and the data are still valid for the training purposes of the auto-encoder, which will be later addressed in this section.

#### 2.4.2. Hyperparameter Tuning and the Auto-Encoder

In order to adjust the parameters that are not directly learned within the neural network training, an exhaustive search generated candidates from a grid of parameters (*grid-search*) [39,40]. This grid consisted of different values for each parameter, such as epochs, learning rate, and loss function. In order to fit the model to the dataset, all the possible combinations of our grid were evaluated, and the best combination was chosen according to a specific metric: in our case, accuracy. 

After performing the *grid-search* method and evaluating the auto-encoder outputs, the chosen hyperparameters during the training phase of the auto-encoder were the mean squared error as the loss function, learning rate α=0.0001, and training for 2000 epochs. The hidden layer neurons used a rectified linear unit activation function to generate the latent space encodings. The output layer neurons used a hyperbolic tangent activation function, given that some reconstructions could present values between −1 and 1. 

A stratified cross-validation of the 20 original scaled features was implemented to obtain the training and a test subset (with a ratio of 80:20, respectively). Afterward, the auto-encoder training was carried out using only the training subset, leaving the test subset out of the training phase. 

In our case, in order to have more certainty that the auto-encoder architecture had learned adequate latent space representations, the test subset, which was never seen by the auto-encoder during its training phase, was used as input for the encoder. The encoder then returned, as an output, latent space representations $C_{k=\{1,\ldots ,10\}}$ that were later used to feed the decoder to generate the reconstructions ${\hat{L}}_{k=\{1,\ldots ,20\}}$. To be more concise, the metrics $R^{2}$ and *MSE* were used to compare the decoder reconstructions ${\hat{L}}_{k=\{1,\ldots ,20\}}$ against the original encoder inputs $L_{k=\{1,\ldots ,20\}}$. Comparing the inputs against the outputs is a way to ensure that the auto-encoder was trained properly. This means that the coefficient of determination, $R^{2}$, via Equation (4) of each biomechanical indicator, $L_{i}$, should be close to 1, as the reconstructions computed by the decoder should have a high level of correlation with the inputs used in the encoder. The mean square error via Equation (7) should be close to 0 as the difference between the inputs and reconstructions of the auto-encoder should be a minimum. However, if a random permutation is applied to the latent space encodings, $C_{k}$, the decoder will not be able to accurately reconstruct the inputs used for the encoder, meaning that both $R^{2}$ and *MSE* will have irregular values.

As depicted in Table 7, the first two columns correspond to the $R^{2}$ and *MSE* values of each feature. With a range that goes from −∞ to 1, the first column $R^{2}$ is close to 1, as is expected in most of the features, and *MSE* is close to 0 due to the high correlation between the inputs and reconstructions in most cases. The next two columns, $R^{2\prime }$ and *MSE*′, correspond to the same metrics but are computed after a random permutation of the latent space representations $C_{k=\{1,\ldots ,10\}}$; as expected, these two metrics now indicate there is no correlation at all between the inputs and the reconstructions of the auto-encoder.

#### 2.4.3. Distance-Based Algorithm Optimization

In this section, different adjustments made to the assessment estimation module were considered to achieve the final evaluation; in this case, KNN, which is widely used [41,42], was selected. This lazy learning algorithm has the advantage that it can be used for classification and regression (KNNR) purposes. In this sense, we can still give a discrete and continuous evaluation for each new patient. 

**Figure 8:**
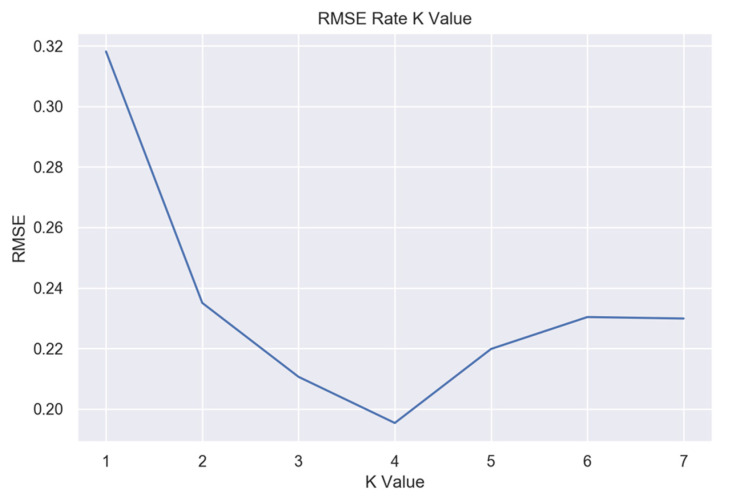
Elbow method for optimal K value in KNN.

Various KNN instances with different *K* values were trained to determine the optimal K value for the nearest neighbor algorithm. Keeping track of the root mean squared error (RMSE) for each instance, the lowest error was found at *K* = 4, as Figure 8 presents. 

As another optimization method, the KNN as a classifier has a variation that assigns weights proportional to the inverse of the distance from the query point. In this variation of the KNN, closer neighbors of a query point will have a greater influence than further away neighbors by Equation (8).

(8) $$y^{\prime }=\underset{v}{\mathrm{argmax}}\sum_{i=1}^{k}w_{i}\;*\;I(v=y_{i})$$

where

$y^{\prime }:$ is the predicted class of the query point;
*v:* represents the class labels;
$w_{i}:$ is the weight computed by Equation (9).

(9) $$w_{i}=\frac{1}{{d(x_{q},x_{i})}^{2}}$$

where

$x_{q}:$ is the new query point;
$x_{i}:$ is the existing point.

For the discrete evaluation, the results of different combinations are depicted in Table 8, where the base KNN, without any previous optimization methods, presented an overall accuracy of 0.72. Precision and recall of categories 2 and 3, which correspond to mild and moderate stages of the disease, showed bad scores. In this sense, the reference considered was a regular KNN without oversampling the 20 original features, which presented an overall accuracy of 72% and poor results in the smaller classes regarding precision and recall. This was expected due to the original dataset imbalance. 

After performing the different optimization methods discussed in the previous sections, the performance increased. By using the oversampled data, the trained auto-encoder using the chosen hyperparameters and the weighted variation of the KNN, there was a considerable increase in the results. Table 8 shows that overall accuracy increased by 0.17, reaching 0.89. Precision and recall also improved in each category, particularly for categories 2 and 3.

Another possible configuration of the KNN is the K-Nearest Neighbors Regression (KNNR). This variation is commonly used in cases where the data labels are continuous rather than discrete variables and are computed using Equation (10). A simple KNNR uses uniform weights: each point in the local neighborhood attributes the same importance to all neighbors [43]. However, KNNR can assign weights proportional to the inverse of the distance as in the classification. This means that nearby points contribute more to the regression than distant points.

(10) $$y^{\prime }=\frac{\sum_{i=1}^{n}\left(w_{i}\;*\;x_{i}\right)}{\sum_{i=1}^{n}w_{i}}$$

where

$y^{\prime }:$ is the predicted value of the query point;
$x_{i}:$ is the latent space representation;
$w_{i}:$ is the weight computed by Equation (9).

In this sense, Table 9 depicts the metrics of the mean squared error (*MSE*) and $R^{2}$ of KNNR using the test set. Good scores for both *MSE* and $R^{2}$ were obtained. This confirms that the latent space encodings generated by the encoder were good representations of the original data.

## 3. Results

Patients can present affectations not evaluated by the UPDRS, such as wobble during the hand movements and the speed change rate during the different stages of an exercise (see Figure 3). These affections are very difficult to assess visually and it is even more complex to monitor their evolution in regular patient consultations. The pronation and supination assessment in Parkinson’s patients, as presented in [20], evaluates motor affectations not covered by the MDS-UPDRS. The referenced study used 12 new indicators that were not considered in [19], which used eight. After a feature selection process, the selected biomechanical features (12 new indicators initially) were used in a new model [20], which integrated a fuzzy inference system, an analytic hierarchy process (AHP) [9], and assessments based on MDS-UPDRS only [19].

However, this paper processes all 20 biomechanical indicators (including the 12 new indicators) using a ***latent space representation*** to obtain the same performance reported in [5] and other additional advantages. This work continues using the MDS-UPDRS Guidelines, with discrete and continuous scores, evaluating extended motor affections; medical experts’ knowledge was integrated during system development and programming. However, an adaptable and scalable method was proposed to reduce complexity during potential redesign needs if new assessment guidelines appear or if other biomechanical indicators need to be incorporated. Likewise, the computer method may be feasible for evaluating the items of the MDS-UPDRS, such as kinetic tremors, hand tremors, leg agility, rest tremor, and gait, among others. 

Biomechanical evaluations of Parkinson’s patients performed by computer systems and based on measurements have the possibility to quantify, with great accuracy, behaviors that are impossible to detect visually, allowing for better monitoring of the evolution of patients. Under the same conditions and states of the patients, their results are repeatable and do not depend on subjective aspects or the evaluator’s expertise. The systems presented have been widely accepted since the medical experts’ knowledge is incorporated into their development and programming.

For the discrete evaluation, in the work presented by the authors of [20], three expert clinicians rated each patient’s pronation and supination movements. In order to make an objective comparison, each expert’s ratings were considered the desired target and compared to the other two expert’s ratings with a cross-validation method. 

In this paper, after performing hyperparameter tuning and other optimization methods, the best-obtained results for the classification were obtained and are depicted in Table 10. It can be seen that the results of the adaptive method outperformed both the reference presented in Table 4 and the performance of the expert clinicians in Table 3.

All the evaluations made by the expert clinicians in [20] followed the MDS-UPDRS guidelines. In this sense, all of them were discrete values ranging from 0 to 4 (evaluations with a value of 4 (severe) were not observed in this dataset, as this score means that the patient could not perform any movement at all).

Another way to visualize the method’s performance is through a receiver operating characteristics (ROC) curve, which helps to identify how well our method can distinguish between the classes. ROC is a performance metric for classification models. It plots the true positive rate (TPR) against the false positive rate (FPR) at different classification thresholds. The TPR represents the percentage of positive instances correctly classified as positive, in our case, patients with PD whose severity stage was correctly identified by our model. In contrast, the FPR represents the percentage of negative instances incorrectly classified as positive; in other words, it measures the ratio between the number of patients’ PD severity stages that are mistakenly classified and the total number of actual patients’ PD severity stages in the dataset. A model with a higher TPR and a lower FPR performs better, achieving a higher area under the ROC curve (AUC).

**Figure 9:**
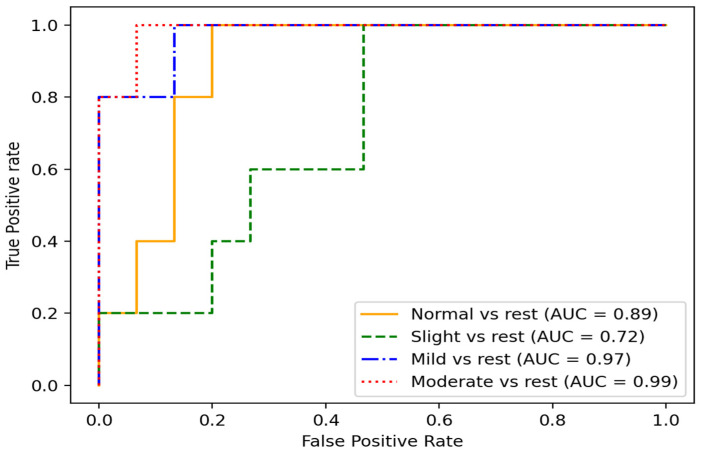
ROC curves and AUC of different stages of disease in PD patients.

As shown in Figure 9, for the multiclass problems, the ROC curves were plotted using one class vs. the rest. In this sense, the area under the ROC curve (AUC) was calculated for each class individually.

The closer the AUC was to 1, the better the method was at accurately distinguishing between classes. As expected, the mild and moderate stages of the disease tended to be easily identified due to noticeable motor affectations during pronation and supination hand movement. 

The evaluation presented in [20] was used as the reference (AHP) to better understand the scores computed by the KNNR. After a mapping process, a subset of the test set was obtained and is shown in Table 11. The first column depicts patient observation. The second column shows the score, as given by the decision-making process used in [20]. The next column gives the continuous evaluation of the current method (KNNR). The last three columns show the scores given by the expert clinicians during the motor assessment. 

**Figure 10:**
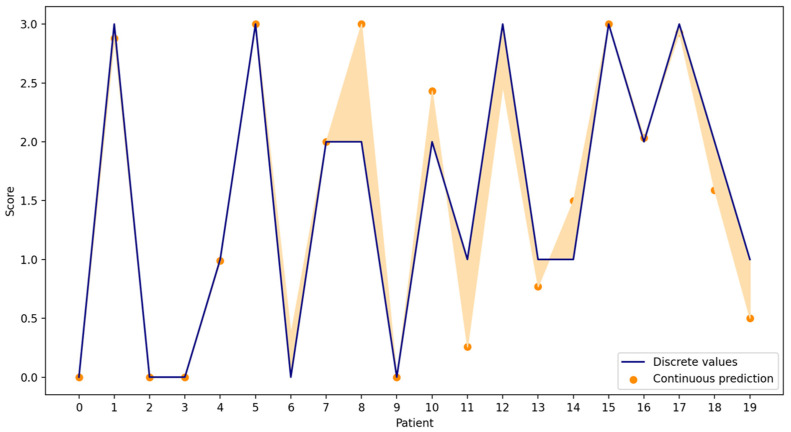
KNNR results vs. discrete values for the test set.

In the proposed method, as depicted in Figure 10, the KNNR continuous scores of the test subset are represented by the orange dots, while the discrete values of the same test set are represented by the blue line. As seen in Figure 10, the scores are very close to each other. This validates that both evaluations were similar for the discrete (KNN) and continuous (KNNR) conditions. 

Our team’s studies have been based on the MDS-UPDRS due to its wide use and international acceptance. The previous statement does not imply that the computer models consider those aspects not included in the MDS-UPDRS because it is a scale that is essentially oriented toward its use by human evaluators. Likewise, medical experts have participated in developing, validating, and verifying such results, facilitated by using models based on human reasoning, where possible. However, the general complexity can increase significantly in the models based on human reasoning as the input data increase. Therefore, the model depicted in Figure 4a) tries to maintain the advantages of a model based on human reasoning, labeling the gained clinical knowledge in Module 2. In this stage, the examiners label the latent space of the *j* dimension based on each original observation without reductions $L_{1–20}$.

## 4. Discussion

Our research team used fuzzy inference models in several previous works [3,11,19,20,44,45,46] regarding biomechanical analysis in Parkinson’s patients due to their proven efficiency and because they allow for multidisciplinary collaboration between clinicians and engineers during their design, research, verification, and use in clinical environments. Fuzzy logic provides an inference tool to represent human reasoning procedures in knowledge-based systems. The fuzzy logic theory offers a mathematical framework for modeling the uncertainty of human cognitive processes, which is highly recommended in many applications, including medical evaluations. However, when there might be changes in the expert’s evaluation criteria and the accepted medical guidelines, the membership functions and rules must be redesigned. Therefore, we present this machine learning method that can quickly adapt to new evaluation criteria and modified or expanded medical guidelines by relabeling the original observations (see Figure 4a) and using a lazy learning method (see Figure 4b) without the need for a complete model redesign. 

This project allows for a more expanded assessment than other works related to the motor evaluation of pronation and supination hand movements. In [47], the J48 algorithm was used and was found to be suitable for distinguishing between the categories (0–4) that the MDS-UPDRS states. Our work used 20 features before a latent space transformation for a more in-depth evaluation. These 20 features provide a greater characterization of upper limb motor impairments, including affectations not considered by the MDS-UPDRS, like the unsteady oscillation of pronation and supination movements. 

Other works present continuous scores, such as in [48], where an index to calculate bradykinesia (BKI) is proposed to evaluate the movement of the upper limbs. However, this index is not strictly attached to the evaluation guidelines stated in the MDS-UPDRS for pronation and supination hand movements. 

Although pronation and supination hand movements were included in [26], only the classification between healthy control subjects and patients with PD was made. In contrast, our approach allows for placing each patient within a stage of Parkinson’s disease based on the MDS-UPDRS. Likewise, a numerical indicator with two precision decimal places is presented to quantify the progress of the disease over time and its possible correlation with palliative treatments.

In the study presented by [49], several hand movements were measured, including pronation-supination, to objectively quantify bradykinesia, tremors, and rigidity in patients with PD. In contrast to our work, the parameters were calculated over small time frames, and certain aspects, such as hesitation in the movements or a decrease in amplitude over time, could not be included. Besides, encompassing several affectations makes it challenging to deepen the pronation and supination analysis. For example, the MDS-UPDRS has four items to evaluate hand affectations: hand movements are covered in item 3.5; pronation and supination hand movements are covered in item 3.6; postural tremor in the hands in item 3.15 [11], and kinetic tremor in the hands, which is another type of affectation mentioned in the scale, is covered by item 3.16 [3,46].

The results presented in [20] are perhaps among the most frequently used for comparisons regarding the current proposal. This is because the same experimental data were used for evaluating motor impairments in PD patients, making it suitable for comparisons between different processing methods using the same data.

To illustrate this in detail, observations 52 and 79 correspond to patients with almost no motor affectations during pronation and supination hand movements (Table 11). These observations show that the three expert clinicians rated the patient as normal or as being in a slight stage of severity. Besides the expert’s ratings, both methods (the decision-making process [20] and the current method) rated the patient with a low score, meaning minimal motor impairments. 

Observation 138 shows a patient in a slight stage of the disease, according to most of the expert examiners. For this case, the scores of both [20] and our method (the current method) depict values close to 1. For patients with more severe motor impairments, such as in observations 163 and 218, the scores for both [20] and the current method were very close to the expert’s evaluation, which rated the patients as being in a severe stage.

A comparison between the works presented in [19,20] and the current method is depicted in Table 12. We can see that the current proposal keeps most of the advantages, such as discrete and continuous evaluations. This is important because the continuous scores can give clinicians a more precise idea of each patient’s severity.

The other key aspects of the current proposal are its adaptability and scalability when compared to the other methods. In this sense, if new evaluation guidelines appear, the proposed method can quickly adapt and be retrained. Systems based on fuzzy inference models might require redesigning the membership functions and the fuzzy rules. The aforementioned does not diminish the importance of fuzzy inference systems, which are very valuable in many applications and areas. For example, when the team members are interdisciplinary researchers, both the results validations and the designs are based on the expertise and agreement of each specialist; this is also true for developing explainable expert systems and automatic controls, among others.

**Figure 11:**
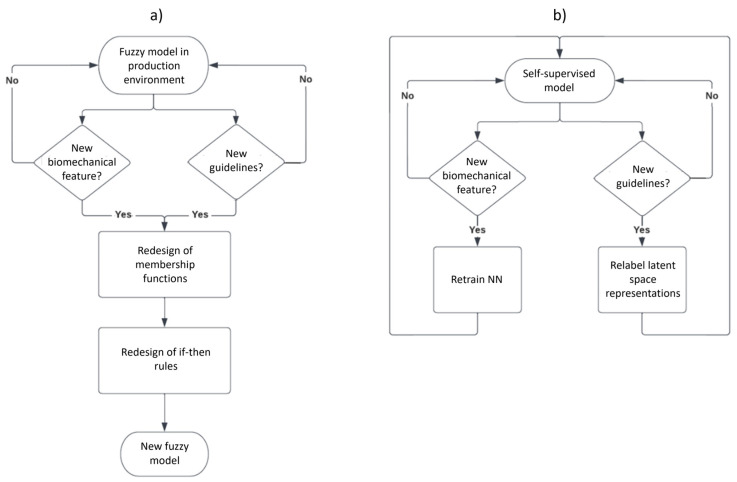
Adaptability and scalability based on new biomechanical features and/or new MDS-UPDRS guidelines. (**a**) Fuzzy inference model. (**b**) Self-supervised model.

To be more explicit, Figure 11 depicts the possible paths one should follow to adapt the method to potential changes. In the case of adding new biomechanical indicators for a fuzzy inference model (Figure 11a), a complete redesign of the membership functions and their rules should be made. In contrast, in a self-supervised model (Figure 11b), only the retraining of the auto-encoder is required to handle the new indicators.

The same scenario applies to the fuzzy inference models (Figure 11a) when new evaluation guidelines appear. In this case, the ranges of the original membership functions can be severely impacted, which will result in the time-consuming task of redesigning the membership functions and their rules.

On the contrary, for a self-supervised model (Figure 11b), the impact of new guidelines is comparatively negligible regarding the method’s adaptability, as the only needed action is to relabel the patient’s evaluation dataset. This will result in relabeling the latent space representations as they are mapped from the original dataset. In our case, a lazy learning algorithm is used for patient assessment, meaning there is no explicit training step; the prediction is adjusted without retraining for a new model, which helps with the adaptability and scalability of our work.

## 5. Conclusions

In this paper, the proposed method analyzes 20 biomechanical indicators, obtaining very good scores when compared to those obtained in the state-of-the-art models, and was verified by expert clinicians during a motor assessment. All this was accomplished while following the MDS-UPDRS guidelines and incorporating the experts’ knowledge into the method.

For a more detailed assessment of each patient, the evaluation stage gives two outputs: a discrete and a continuous score, with a scalable and adaptable method that requires less effort during both design (or redesign) and implementation. Both scores are still strictly attached to the MDS-UPDRS guidelines. While the discrete score gives the severity stage of each patient (which is what expert clinicians now do), the continuous score allows for a more detailed evaluation and clinical follow-up of patients with PD, even if they are in the same stage.

The proposed method can be quickly scaled and adapted when new evaluation conditions appear. In this sense, it can be adapted if the guidelines used by the experts to evaluate patients change over time, effectively relabeling the patients’ records. Regarding scalability, new biomechanical indicators can be incorporated into a production environment with less effort. In this sense, a complete redesign is unnecessary; only retraining the neural network that transforms the biomechanical indicators to their latent space representation is required.

Finally, the proposed method was implemented and integrated into previously developed software that is already used in clinical environments, significantly reducing the time for each clinical assessment. After acquiring the patient’s raw signals with the help of sensors, they are processed by the previously developed software to extract the biomechanical features, and the assessment is computed by the current method almost instantly, given that the last part of the process has a computational complexity of O(kn). 

Regarding research limitations, this computer method, which is based on latent space representations of biomechanical indicators, has been proven and verified for pronation-supination hand movements. Future research might include its development, applicability, testing, and validation for other complex items within the MDS-UPDRS, such as 3.9: Arising from the chair, 3.10: Gait, 3.12: Postural stability, and 3.13: Posture, among others. In this sense, new measures are recommended to consider reasonable proportions between healthy volunteers and PD patients, men, women, and different ages. 

## Figures and Tables

**Table 1:** PD patients’ summary.

Characteristics	PD Patients	Healthy Patients
Age Range	48–83 years	23–63 years
Range of Years with a PD diagnosis	1–20 years	-
Age Average	67.4 years	31.28 years
Age Standard Deviation	9.3 years	14.1 years
Male Subjects	34	5
Female Subjects	23	3

**Table 2:** Description of biomechanical features computed in the pronation and supination sequence. Sexagesimal degree (°).

Key	Description	Units
	**Amplitude related features**	
$L_{1}$	Mean amplitude	∘
$L_{2}$	Amplitude’s standard deviation	∘
$L_{6}$	Amplitude decrements in the first stage of the sequence	∘
$L_{7}$	Amplitude decrements in the second stage of the sequence	∘
$L_{8}$	Amplitude decrements in the last stage of the sequence	∘
$L_{11}$	Median amplitude	∘
$L_{12}$	The interquartile range of amplitude	∘
	**Velocity related features**	
$L_{3}$	Mean velocity	∘/s
$L_{4}$	Velocity’s standard deviation	∘/s
$L_{13}$	Median velocity	∘/s
$L_{14}$	The interquartile range of velocity	∘/s
$L_{15}$	Rate of velocity decrement in the first stage of the sequence	-
$L_{16}$	Rate of velocity decrement in the second stage of the sequence	-
$L_{17}$	Rate of velocity decrement in the last stage of the sequence	-
$L_{18}$	The slope of velocity decrement in the first stage of the sequence	-
$L_{19}$	The slope of velocity decrement in the second stage of the sequence	-
$L_{20}$	The slope of velocity decrement in the last stage of the sequence	-
	**Anomalies during movement’s sequence**	
$L_{5}$	Halts & hesitations	−
$L_{9}$	Unsteady oscillation in the *x*, *y*, and *z* axes	cm
$L_{10}$	Unsteady oscillation in the *x* and *y* axes	cm

**Table 3:** Cross-validation scores of three expert clinicians [20].

	Expert 1	Expert 2	Expert 3
**Expert 1**	1	0.72	0.7
**Expert 2**	0.72	1	0.74
**Expert 3**	0.7	0.74	1

**Table 4:** KNN metrics report.

	Category	Precision	Recall	F1-Score
**KNN**	0	0.8	0.62	0.7
	1	0.73	0.92	0.81
	2	0.5	0.17	0.25
	3	0.5	0.67	0.57

**Table 5:** KNNR metrics report.

	*MSE*	$R^{2}$
**KNNR**	0.05	0.86

**Table 6:** Proportions of categories before and after oversampling.

Category	Before Smote	After Smote
**0**	27%	25%
**1**	53%	25%
**2**	13%	25%
**3**	7%	25%

**Table 7:** $R^{2}$ and *MSE* values of each feature comparing the original inputs vs. decoder reconstructions after latent space representations are randomly shuffled.

	$\mathrm{Without}\;\mathrm{Random}\;\mathrm{Permutation}\;C_{k}$		$\mathrm{With}\;\mathrm{Random}\;\mathrm{Permutation}\;\mathrm{of}\;C_{k}$	
Feature	$R^{2}$	*MSE*	$R^{2\prime }$	*MSE′*
***L1***	0.952	0.001	−0.379	0.057
***L2***	0.891	0.002	−1.882	0.067
***L3***	0.951	0.002	−0.579	0.073
***L4***	0.927	0.001	−1.351	0.058
***L5***	0.945	0.001	−1.006	0.066
***L6***	0.55	0.005	−1.953	0.036
***L7***	0.866	0.002	−1.945	0.046
***L8***	0.801	0.004	−0.778	0.037
***L9***	0.956	0.001	−0.689	0.059
***L10***	0.961	0.001	−0.859	0.078
***L11***	0.945	0.002	−0.559	0.066
***L12***	0.899	0.002	−1.578	0.076
***L13***	0.964	0.001	−0.678	0.075
***L14***	0.915	0.002	−1.412	0.071
***L15***	0.502	0.001	−2.056	0.008
***L16***	0.866	0.001	−3.146	0.053
***L17***	0.747	0.003	−1.508	0.035
***L18***	0.704	0.003	−1.542	0.026
***L19***	0.742	0.003	−1.516	0.034
***L20***	0.713	0.002	−2.156	0.031

**Table 8:** KNN classifier results.

			Category	Precision	Recall	F1-Score	Overall Accuracy
**Original dataset (20 features)**	Original data	KNN	0	0.8	0.62	0.7	0.72
			1	0.73	0.92	0.81	
			2	0.5	0.17	0.25	
			3	0.5	0.67	0.57	
		KNN-Weighted	0	0.78	0.54	0.64	0.65
			1	0.69	0.83	0.75	
			2	0.25	0.17	0.2	
			3	0.5	0.67	0.57	
	Oversampled data	KNN	0	0.7	0.96	0.81	0.84
			1	0.92	0.48	0.63	
			2	0.88	0.92	0.9	
			3	0.92	1	0.96	
		KNN-Weighted	0	0.77	0.96	0.85	0.86
			1	0.93	0.52	0.67	
			2	0.85	0.96	0.9	
			3	0.92	1	0.96	
**Latent Space (10 dimensions)**	Original data	KNN	0	0.77	0.77	0.77	0.76
			1	0.78	0.88	0.82	
			2	0.67	0.33	0.44	
			3	0.67	0.67	0.67	
		KNN-Weighted	0	0.75	0.69	0.72	0.7
			1	0.74	0.83	0.78	
			2	0.33	0.17	0.22	
			3	0.5	0.67	0.57	
	Oversampled data	KNN	0	0.73	1	0.84	0.87
			1	0.88	0.56	0.68	
			2	0.92	0.92	0.92	
			3	1	1	1	
		KNN-Weighted	0	0.82	0.96	0.88	0.89
			1	0.89	0.64	0.74	
			2	0.85	0.96	0.9	
			3	1	1	1	

**Table 9:** KNNR results.

		*MSE*	$R^{2}$
**Latent space encodings**	KNNR	0.19	0.84
	KNNR weighted	0.14	0.88

**Table 10:** KNN classifier results after performing the hyper-parameter tuning and optimization.

Category	Precision	Recall	F1-Score	Overall Accuracy
*Normal*	0.82	0.96	0.88	0.89
*Slight*	0.89	0.64	0.74	
*Mild*	0.85	0.96	0.9	
*Moderate*	1	1	1	

**Table 11:** A subset of continuous scores in the test set.

# of Patient Observation	Method in [20]	Current Method	Expert 1	Expert 2	Expert 3
**163**	2.99	**2.88**	3	3	2
**218**	2.81	**2.92**	3	3	3
**79**	0.34	**0.36**	0	1	1
**72**	1.77	**2.03**	2	2	2
**138**	0.98	**0.99**	2	1	1
**52**	0.36	**0.25**	1	0	0

**Table 12:** Comparison of models presented in [19,20] vs. the current model.

	Method Proposed in [19]	Method Proposed in [20]	Current Method
Follows MDS-UPDRS Guidelines	✓	✓	✓
Evaluates extended motor affectations		✓	✓
Discrete evaluation	✓	✓	✓
Continuous evaluation	✓	✓	✓
Ease of interpretability	✓	✓	✓
Integrates expert knowledge	✓	✓	✓
Adaptability			✓
Scalability			✓
Reduced complexity during design and redesign			✓

## Data Availability

The data presented in this study are available upon request from the corresponding author.
